# In Situ Thrombosis of Small Pulmonary Arteries in Pulmonary Hypertension Developing after Chemotherapy for Malignancy

**DOI:** 10.1155/2015/230846

**Published:** 2015-01-27

**Authors:** Kay Maeda, Yoshikatsu Saiki, Shigeo Yamaki

**Affiliations:** ^1^Japanese Research Institute of Pulmonary Vasculature, 2-2-26 Seikaen Aoba-ku, Sendai 982-0262, Japan; ^2^Division of Cardiovascular Surgery, Tohoku University Graduate School of Medicine, 1-1 Seiryo-machi, Aoba-ku, Sendai 980-8574, Japan

## Abstract

A few reports have provided histopathological insight into pulmonary hypertension developing after antitumor chemotherapy. In general, plexogenic pulmonary arteriopathy is a commonly observed finding in patients with severe pulmonary hypertension. We herein report a novel pathological finding that may characterize the histopathological change occurring in patients with pulmonary hypertension after chemotherapy for malignancy. Lung biopsy or autopsy was performed in 7 patients with pulmonary hypertension that developed during or after chemotherapy between 2006 and 2013 to examine the pulmonary vascular changes or to determine the cause of death. Pathological findings included in situ thrombosis in the small pulmonary arteries in 4 of 7 patients. In 2 of 4 patients, pulmonary hypertension was controlled by anticoagulants and antithrombotic agents. One patient who had organized thrombi attained spontaneous remission with oxygen therapy. The other patient died of sudden cardiopulmonary arrest during chemotherapy. Autopsy showed complete occlusion of the peripheral small pulmonary arteries and veins by thrombi. These results demonstrate that in situ thrombosis in the small pulmonary arteries could cause pulmonary hypertension after chemotherapy.

## 1. Introduction

Pulmonary hypertensive disease can develop after chemotherapy for various malignant tumors and becomes lethal in immunocompromised patients. A few case reports have implicated a probable relationship between chemotherapy and subsequent pulmonary hypertension (PH) [1–3]. However, there have been few reports on pathological findings that are characteristic to this devastating sequela [4].

To date, we have performed a significant number of lung biopsies for diagnoses of severe PH associated with congenital and acquired heart diseases [5]. We reported distinctive pulmonary vascular changes even in relatively rare diseases associated with PH, such as chronic thromboembolic pulmonary hypertension (CTEPH), pulmonary venoocclusive disease (PVOD), idiopathic pulmonary arterial hypertension (IPAH), and portosystemic venous shunt (PSVS) [6–10]. In patients with severe PH, plexogenic pulmonary arteriopathy generally develops in the small pulmonary arteries [11].

In the present study, we demonstrate that in situ thrombosis in the small pulmonary arteries is one of the causative mechanisms for PH associated with chemotherapy.

## 2. Materials and Methods

Between 2006 and 2013, 7 patients who developed PH after chemotherapy for various malignancies were histopathologically examined at our research institute. Lung biopsies (3 cases) and autopsies (4 cases) were conducted to investigate the cause of unexplained PH after chemotherapy or to determine the cause of death, in detail, in terms of histopathological characteristics. The Heath-Edwards (HE) classification was used to assess the arterial changes [12].

Lung tissue was obtained from a lobe of the lung and was fixed in 10% formalin. In each case, 30 semiserial histological sections at 50 *μ*m intervals, each 3 *μ*m thick, were prepared as previously described [5]. Then, modified Elastica–Goldner staining was performed for the histopathological analysis [13]. All protocols were conducted in compliance with the Helsinki Declaration. They were also approved by the Ethical Committee of the Japanese Research Institute of Pulmonary Vasculature and each referred hospital. Informed consent was obtained from all patients.

## 3. Results

The clinical and pathological characteristics of patients who developed PH after chemotherapy are presented in Table 1. Most patients (6/7) were young children (2–6 years old), including 4 lethal cases. The time interval between chemotherapy and the onset of PH varied from a few days to years. Histopathological analysis revealed that the cause of unexplained PH was in situ thrombosis in the small pulmonary arteries in 4 of 7 patients (cases 1–4). In the other 3 patients (cases 5–7), the pathological diagnoses were PVOD, IPAH, and plexogenic arteriopathy. In case 3, congenital partial protein C deficiency, which causes a clotting disorder, was detected after lung biopsy as the result of intensive examination to investigate the cause of thrombi. However, the others (cases 1, 2, and 4) had no thrombotic diathesis. The clinical findings of patients (cases 1–4), who were pathologically diagnosed with in situ thrombosis in small pulmonary arteries, are shown in Table 2.

**Figure 1:**
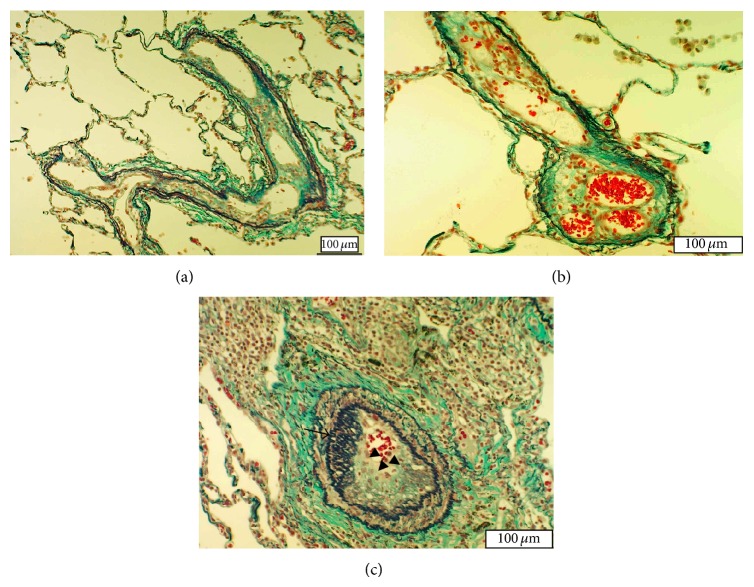
Histopathological findings of lung tissue section obtained from case 1 visualized by Elastica-Goldner staining. (a) Small pulmonary artery containing old thrombi. The lumen is separated into several channels by recanalization of organized thrombi. (b) Pulmonary vein with the recanalized thrombus separating the lumen into 3 channels. (c) Small pulmonary artery (200 *μ*m in diameter) with moderate medial thickening and longitudinal smooth muscle proliferation inside the lumen (arrow). The thrombus is shown in the lumen (triangular arrow).

A 46-year-old man (case 1) underwent surgery and chemotherapy for primary testicular malignant lymphoma at the age of 38 years and remained in remission after treatment. He began to experience shortness of breath on exertion and was hospitalized due to suspicion of diffusive lung disease. Detailed examination showed no evidence of cancer recurrence. Computed tomography of the chest demonstrated ground-glass opacity. No myocardial disease was noted on the echocardiogram. Right catheterization showed tapering vascular shadows of both upper lungs, with a mean pulmonary artery pressure (PAP) of 30 mmHg. Transbronchial lung biopsy revealed mild inflammatory cell infiltration but did not yield a definite diagnosis. Therefore, video-assisted thoracic surgery was performed. Histopathology revealed long-term and extensive in situ thrombosis in the small pulmonary arteries (Figure 1(a)) and pulmonary veins (Figure 1(b)). Both the small pulmonary arteries and veins exhibited recanalization of organized fibrotic thrombi, with the lumen separated into several channels. Some small pulmonary arteries (>100 *μ*m in diameter) showed luminal narrowing and occlusion by longitudinal smooth muscle (Figure 1(c)). There was mild inflammatory cell infiltration, but no evidence of vasculitis. Laboratory data confirmed the absence of vasculitis syndrome or coagulation disorder. The respiratory symptoms gradually improved with oxygen therapy.

**Figure 2:**
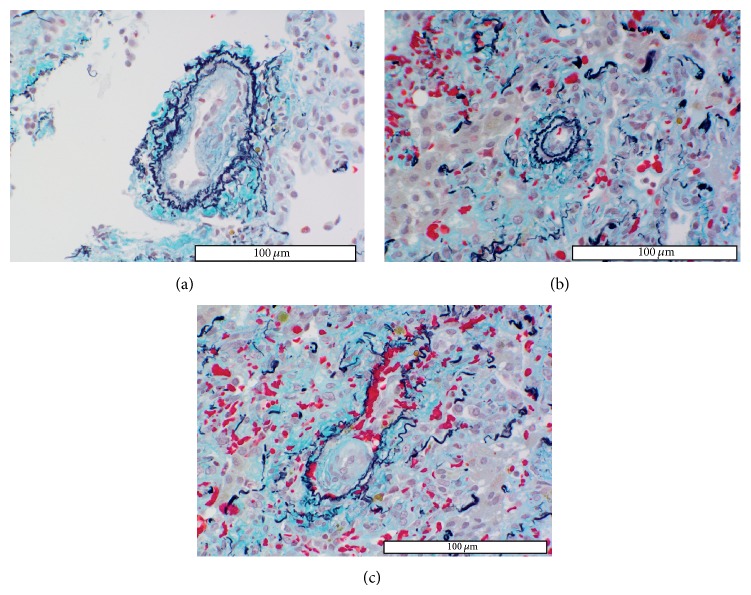
Histopathological findings in small pulmonary arteries and pulmonary veins of case 2. (a) Peripheral small pulmonary artery with newly formed eccentric intimal fibrous proliferation considered to be a thrombus, because intimal lesions usually form concentric layers in plexogenic arteriopathy. (b) The peripheral small pulmonary artery (40 *μ*m in diameter) shows complete occlusion by a thrombus. (c) Most small pulmonary veins are almost occluded by newly formed thrombi.

A 6-year-old girl (case 2) presented with abdominal distention and abdominal pain. A biopsy specimen of the abdominal wall showed primary right adrenal neuroblastoma. Therefore, chemotherapy for neuroblastoma was initiated according to the protocol of the inpatient facility. After the fourth course of chemotherapy, tumorectomy was performed, followed by autologous peripheral blood stem cell transplantation (auto-PBSCT) and high-dose chemotherapy. Soon after completion of the second auto-PBSCT and high-dose chemotherapy, the patient exhibited polypnea and hypoxemia. Chest radiographic examination revealed moderate pleural effusion. As assessed by echocardiography, mean PAP was 40 mmHg. Hypoxia was improved by the administration of diuretics. However, 1 week later, the patient exhibited polypnea, tachycardia, aggravated hypoxemia, and chest pain. Echocardiography showed PH (mean PAP = 70–75 mmHg) and right ventricular enlargement. Mean PAP decreased to 55 mmHg under nitric oxide (NO) inhalation but increased again after NO withdrawal, despite the administration of sildenafil and epoprostenol. Therefore, lung biopsy was performed for suspected PVOD. The small pulmonary arteries (>150 *μ*m in diameter) showed no intimal proliferation but severe medial thickening (HE classification: I). Pathological findings revealed that small pulmonary arteries and veins (<30–50 *μ*m in diameter) were almost completely occluded by newly formed thrombi not yet organized (Figures 2(a)–2(c)). The lung parenchyma presented atelectasis and cellular thickening of the alveolar wall. The patient is currently followed-up with additional administration of anticoagulants.

**Figure 3:**
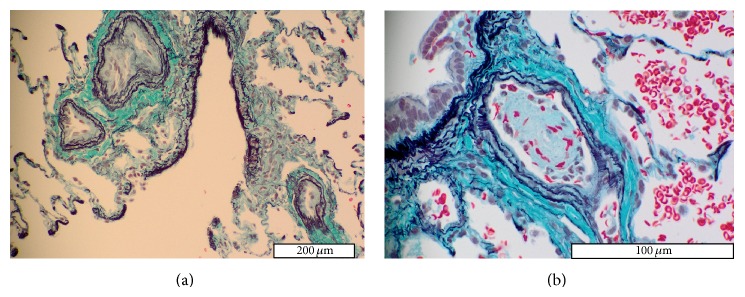
Histopathological findings in small pulmonary arteries and pulmonary veins of case 3. (a) Almost all small pulmonary arteries have mildly thickened media and are occluded by thrombi. (b) Peripheral small pulmonary artery showing almost completely occluded by a newly formed thrombus. Most thrombi shown in small pulmonary arteries are in the process of being formed.

A 2-year-old girl (case 3) was diagnosed with acute lymphocytic leukemia (ALL) at the age of 5 months. She received high-dose chemotherapy and umbilical cord blood transplants. After the second transplant, the patient had a documented PH crisis at the age of 22 months. The respiratory symptoms were effectively relieved by NO inhalation, followed by sildenafil and beraprost administration. Echocardiography showed PH (mean PAP = 40–50 mmHg), without any myocardial disease. Therefore, PAH was suspected based on the clinical findings. Lung biopsy was performed to verify the diagnosis. Microscopically, the small pulmonary arteries showed mild medial thickening but no intimal proliferation (HE classification: I). However, thrombi at varying stages were found in almost all peripheral small-sized pulmonary arteries (Figure 3(a)). Most thrombi were still in the process of formation (Figure 3(b)). In contrast, the pulmonary veins and respiratory system appeared normal. The detailed examination to investigate the cause of these thrombi was carried out based upon the above described lung biopsy findings, and partial congenital protein C deficiency was detected. PH was successfully controlled with anticoagulants and antithrombotic agents.

**Figure 4:**
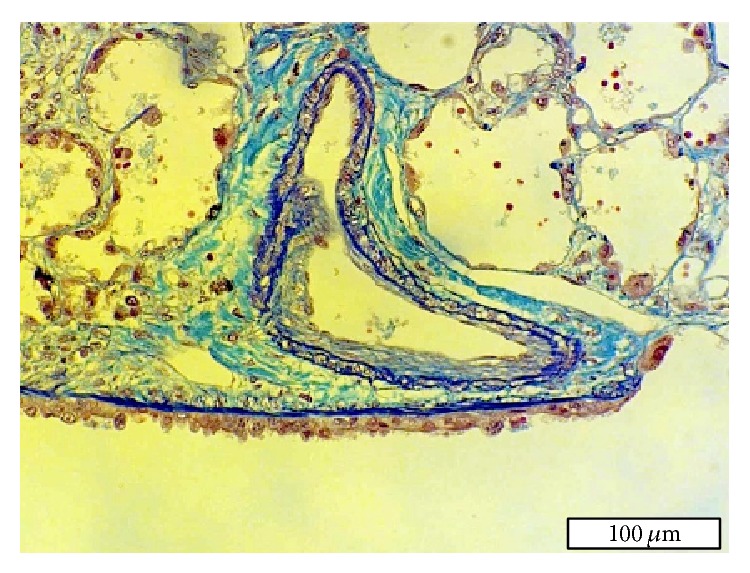
Histopathological findings in a small pulmonary artery of the right lung of case 4. This small pulmonary artery has moderate medial thickening and eccentric intimal fibrous proliferation (thrombus).

A 2-year-old boy (case 4) with rhabdomyosarcoma started receiving chemotherapy at the age of 17 months, and he developed hepatic venoocclusive disease during treatment. At the age of 24 months, he became dyspneic with hypoxemia. Mild PH was detected on the electrocardiogram and echocardiogram. After a few weeks of oxygen therapy, he experienced sudden chest-abdominal pain and died of sudden cardiopulmonary arrest at the hospital at the age of 26 months. Postmortem examination of the lungs was performed to determine the cause of death. The small pulmonary arteries (>200 *μ*m in diameter) showed moderate medial thickening without intimal lesion (HE classification: I). Some small pulmonary arteries (<100 *μ*m in diameter) and pulmonary veins showed intimal fibrous proliferation (Figure 4). They were considered to be thrombi because of the eccentric intimal thickening. Small peripheral pulmonary arteries (<50 *μ*m in diameter) showed complete occlusion by thrombi.

## 4. Discussion

This is the first study reporting in situ thrombosis in the small pulmonary arteries of patients who developed PH after chemotherapy. In contrast, several studies have reported PH due to PVOD after stem cell transplantation or chemotherapy [1–3]. We also diagnosed in 1 patient (case 5) that the cause of unexplained PH developing after chemotherapy was PVOD [4]. The histopathology of PVOD typically shows extensive occlusion of pulmonary veins by intimal fibrosis [14] in the form of concentric layers [6, 7]. The present cases (cases 1–4) exhibited a distinct pattern of thrombosis in pulmonary veins and small pulmonary arteries characterized by localized and eccentric intimal fibrosis. In addition, the fibrosis was associated with recanalization of the organized thrombi by separating the lumen into several channels. In contrast, plexogenic pulmonary arteriopathy, which is typically seen in PH patients, was hardly seen in all patients with in situ thrombosis in the small pulmonary arteries even with high PAP readings.

In situ thrombosis in the small pulmonary arteries is difficult to diagnose from clinical examination due to nonspecific complaints and from laboratory data. Computed tomography typically shows diffuse ground-grass opacities, patchy pulmonary opacity, atelectasis, and pleural effusion. Even with these diagnostic radiological findings, it is difficult to distinguish in situ thrombosis from other pathological conditions that can exhibit similar symptoms, such as PVOD and interstitial pneumonia. In cases 5–7, histopathological examination identified that the causes of PH were PVOD, IPAH, and interstitial pneumonia, whereas clinical findings in these cases were not distinguishable from those with in situ thrombosis. When patients undergoing antitumor chemotherapy present unexplained PH, lung disease and myocardial dysfunction should be ruled out, whereas PVOD and in situ thrombosis should be considered as differential diagnoses. Considering these facts, lung biopsy is necessary for accurate diagnosis for some patients with unexplained PH. In addition, thrombogenic factors should be investigated because some patients may develop thrombotic diathesis [15], as in case 3.

Patient 1 had mild symptoms, likely because the thrombi had developed over several years. Microscopically, organized thrombi with recanalization were found in small pulmonary arteries and veins. The direct cause of PH might be respiratory diseases, because longitudinal smooth muscles appeared under hypoxia. Therefore oxygen therapy was effective in this case. The background of organized fibrotic thrombi, which seem to have been formed a few years previously, might accelerate the bloodstream aggravation in pulmonary circulation, although the symptom was not recognized at the time of thrombosis formation. In cases 2 and 3, anticoagulants and antithrombotic agents were effective treatments for in situ thrombosis. In these cases, sudden PH resistance to pulmonary vasodilators including NO occurred, which indicated that thrombi were forming at that moment. Incidentally, histopathology revealed that small pulmonary arteries were almost completely occluded due to forming or newly formed thrombi. Thus, this wave of new thrombi caused PH to progress rapidly. In such cases, anticoagulants and antithrombotic agents have played a crucial role in survival. Interestingly, in situ thrombosis in small pulmonary arteries is one of the pathological features of PSVS [7]. These patients with PSVS responded very well to prostaglandin E1 treatment. This study suggests that prostaglandin E1 might be an effective therapy for in situ thrombosis in the small pulmonary arteries. Possible hazard to institute anticoagulation and antithrombotic therapy may include lung hemorrhage in the presence of PVOD, which can be avoided by accurate diagnosis with lung biopsy. The most efficient therapy for PVOD is, in fact, currently lung transplant. In contrast, in situ thrombosis can possibly be treated by pharmacotherapy. Regardless, initiation of treatment should not be delayed to avoid a fatal outcome as in case 4. As early diagnosis and treatment is crucial for this disease, clinicians should keep in mind that in situ thrombosis may induce the development of PH. The lung biopsy is currently the sole diagnostic method to identify in situ thrombosis in the small pulmonary arteries.

These studies apparently have significant limitations. First, this report is consisted of small series of selected patients. However, it should be noted that in situ thrombosis in the small pulmonary arteries was detected as the possible cause of PH after chemotherapy in 4 of 7 patients. Secondly, assessment of lung biopsy was carried out only once for each patient; therefore, serial changes could not be elucidated. The precise pathogenesis of in situ thrombosis is small pulmonary arteries still remains unknown.

## 5. Conclusions

The present study demonstrates that in situ thrombosis in the small pulmonary arteries is one of the causative mechanisms for PH developing during chemotherapy. Further research is required to elucidate the pathogenesis of PH after chemotherapy.

## Figures and Tables

**Table 1:** Details of patients with pulmonary hypertension after chemotherapy.

Case	Age, sex	Neoplasm	Treatment for neoplasm	Period from chemotherapy to PH onset	Clinical diagnosis [cause of death]	Pathological findings	Lung specimen
1	46, M	Malignant lymphoma	Surgical resection + chemotherapy	8 years	IP	In situ thrombosis of SPA and veins	Biopsy
2	6, F	NB	Surgical resection + AutoPBSCT (2) + high-dose chemotherapy	7 days	IP, PVOD	In situ thrombosis of SPA and veins	Biopsy
3	2, F	ALL	Umbilical cord blood transplant (2) + high-dose chemotherapy	10 days	PAH, Congenital protein C deficiency	In situ thrombosis of SPA	Biopsy
4	2, M	RMS	Chemotherapy + Proton therapy	During chemotherapy	Hepatic venoocclusive disease [unknown]	In situ thrombosis of SPA and veins	Autopsy
5	5, M	Burkitt's Lymphoma	Chemotherapy	During chemotherapy	PVOD [refractory right HF]	PVOD	Autopsy
6	3, M	MB	Surgical resection + chemotherapy	9 months	s/o PAH, PVOD [IP]	IPAH, IP	Autopsy
7	2, F	AML	Allogeneic bone marrow transplant + high-dose chemotherapy	2 months	s/o PVOD, cGVHD [IP]	Plexogenic arteriopathy, IP	Autopsy

ALL = acute lymphocytic leukemia; AML = acute myelogenous leukemia; AutoPBSCT = autologous peripheral blood stem cell transplantation; cGVHD = chronic graft versus host disease; F = female; HF = heart failure; IP = interstitial pneumonia; IPAH = idiopathic pulmonary arterial hypertension; M = male; MB = medulloblastoma; NB = neuroblastoma; PAH = pulmonary arterial hypertension; PH = pulmonary hypertension; PVOD = pulmonary venoocclusive disease; RMS = rhabdomyosarcoma; SPA = small pulmonary arteries.

**Table 2:** Clinical findings of patients with in situ thrombosis.

Case	Clinical symptoms	Computed tomography	Echocardiography	PAG	Clinical outcome
1	DOE	Ground-glass opacity Diffuse patchy nodular interstitial opacity	Normal	Tapering vascular shadow of both upper lungs, mPAP = 30 mmHg	Oxygen therapy→improved
2	Polypnoea, hypoxemia	Ground-glass opacity Pleural effusion	mPAP = 70–75, 55 mmHg (under NO), RVH	—	Diuretics→severe PH→NO, PDE5i, PGI2→anticoagulants→improved
3	DOE, hypoxemia, polypnoea,	Ground-glass opacity	mPAP = 40–50 mmHg	—	NO, PDE5i, PGI2→anticoagulants, antithrombotic drugs→improved
4	Hypoxemia, sudden chest-abdominal pain	—	mPAP = 50–55 mmHg	—	Oxygen→sudden cardiopulmonary arrest (death)

DOE = dyspnea on exertion; mPAP = mean pulmonary artery pressure; PAG = pulmonary angiography; PDE5i = cGMP-specific phosphodiesterase type 5 inhibitor; PGI2 = prostaglandin I2; PH = pulmonary hypertension; RVH = right ventricular enlargement.
